# A Micro Saddle Coil with Switchable Sensitivity for Local High-Resolution Imaging of Luminal Tissue †

**DOI:** 10.3390/mi7040067

**Published:** 2016-04-21

**Authors:** Tetsuji Dohi, Kousuke Murashige

**Affiliations:** Faculty of Science and Engineering, Chuo University, 1-13-27 Kasuga, Bunkyo-ku, Tokyo 112-8551, Japan; dohi@mech.chuo-u.ac.jp

**Keywords:** micro coil, magnetic resonance imaging (MRI), saddle coil, switchable sensitivity

## Abstract

This paper reports on a micro saddle coil for local high-resolution magnetic resonance imaging (MRI) fabricated by embedding a flexible coil pattern into a polydimethyilsiloxane (PDMS) tube. We can change the sensitivity of the micro coil by deforming the shape of the coil from a saddle-shaped mode to a planar-shaped mode. The inductance, the resistance, and the Q-factor of the coil in the saddle-shaped mode were 2.45 μH, 3.31 Ω, and 39.9, respectively. Those of the planar-shaped mode were 3.07 μH, 3.92 Ω, and 42.9, respectively. In MRI acquired in saddle-shaped mode, a large visible area existed around the coil. Although the sensitive area was considerably reduced in the planar-shaped mode, clear MRI images were obtained. The signal-to-noise ratios (SNR) of the saddle-shaped and planar-shaped modes were 194.9 and 505.9, respectively, at voxel size of 2.0 × 2.0 × 2.0 mm^3^ and 11.7 and 37.4, respectively, at voxel size of 0.5 × 0.5 × 1.0 mm^3^. The sensitivity of the saddle-shaped and the planar-shaped modes were about 3 times and 10 times higher, respectively, than those of the medical head coil at both voxel sizes. Thus, the micro saddle coil enabled large-area imaging and highly sensitive imaging by switching the shape of the coil.

## 1. Introduction

**Figure 1:**
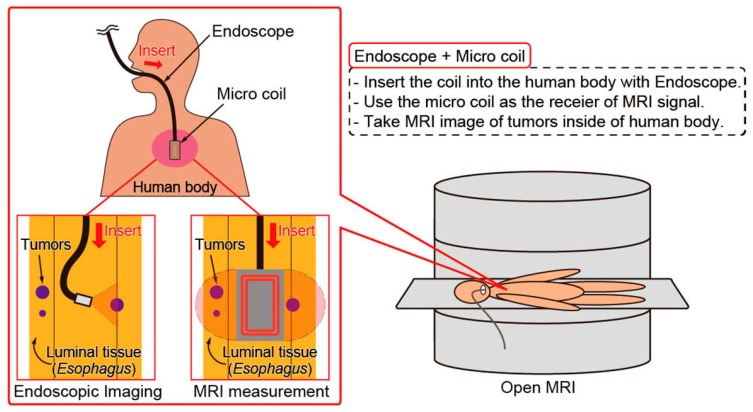
Concept of using the micro coil for magnetic resonance imaging (MRI) inside of the human body. By using a combination of endoscope and the micro coil, not only surface tumors but also small deep tumors in luminal tissue can be detected.

Magnetic resonance imaging (MRI) is a promising method to visualize the inside of a human body from nuclear magnetic resonance (NMR) signal. In recent years, it has been made an attempt to measure the MRI images during surgery for safe and reliable operation [1,2,3]. The endoscopic MRI has also been studied as an attempt of the intraoperative MRI [4,5,6,7,8]. By using MRI endoscopically, as shown in Figure 1, early detection of tumors that occur in deep luminal tissue is expected. Generally, it is difficult to find tumors or cancer cells that have occurred in deep luminal tissue such as the esophagus or the small intestine. Since only surface tissue is observed in the endoscopic imaging, deep tumors cannot be detected by the endoscope. By using a combination of endoscope and MRI, we can acquire the depth of the tumors in addition to detecting the tumors in deep luminal tissues. Furthermore, it is possible to prevent the tumors from cutting away unnecessarily normal tissue.

Although MRI has the advantage of non-invasive imaging, there is a problem of low spatial resolution. Since the NMR signal is drastically reduced in the high-resolution MRI imaging, it is difficult for imaging cross sections of small luminal tissue such as the esophagus. Therefore, many studies on improving the resolution of MRI have been reported [7,8,9,10,11,12,13,14]. The improvement of MRI resolution can be achieved by improving the signal-to-noise ratio (SNR) of the MRI signal. The SNR of the MRI signal is proportional to the number of turns of the MRI signal receiving coil, and in inverse proportion to the diameter of the coil and the 0.5 power of the resistance of the coil [15].

Therefore, high SNR images can be easily acquired by micro coils because micro coils have small diameter, low resistance, and a large number of turns.

As for high sensitive micro coils, the planar micro coils for MRI have been reported [8,9,10,11]. Since the planar coils have many wiring per unit length, the sensitivity of the planar coils is very high, and high-resolution MRI images could be acquired. However, the sensitive area of the planar micro coil is small because the sensitive area of the planar coil is almost the same as the spherical area of same diameter as the coil. Therefore, it is difficult to image the entire area of the luminal tissue. Three-dimensional (3D) micro coils for MRI were also studied [12,13,14]. Since these micro coils have 3D structure, the sensitive area is increased as compared to the planar micro coils. However, since the 3D micro coils have the sensitive area mainly inside of the coil, the 3D micro coil is not suitable for imaging luminal tissue.

In another study, the saddle coils for endoscopic MRI were reported which can take MRI images of luminal tissue [6,16]. The thin-film saddle coil was also reported [17]. Although the saddle coils have the large sensitive area around the coil, the sensitivity of the saddle coil is not high enough for detecting a small tumor. Furthermore, the saddle coil is difficult to fabricate by micromachining because the saddle coil has a complicated 3D structure.

In this study, we propose a micro saddle coil with switchable sensitivity for local high-resolution MRI. The concept and first experimental results were reported at MEMS2015 [7]. Our micro saddle coil adopts two shape modes: saddle-shaped mode and planar-shaped modes. The altered shape of the micro saddle coil changes the sensitive area and sensitivity of the coil. In this way, the micro saddle coil can acquire both large-area as well as highly sensitive MRI images for detecting the tumors in deep luminal tissue.

## 2. Concept and Fabrication

### 2.1. Concept of the Micro Saddle Coil

**Figure 2:**
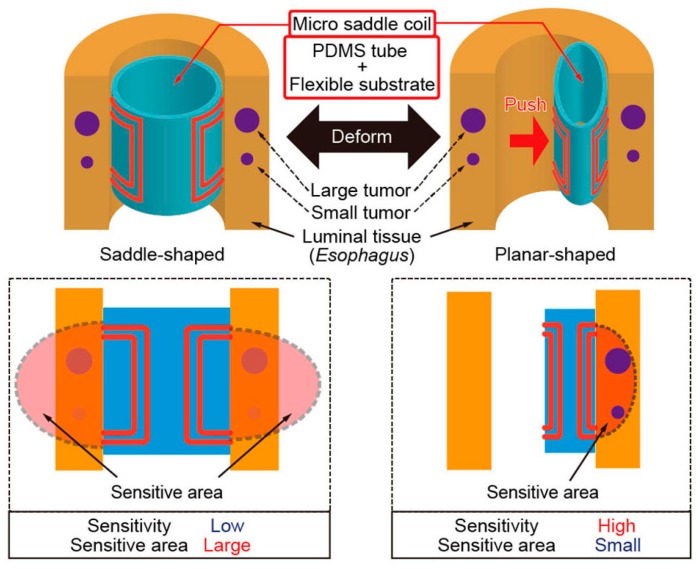
Concept of the micro saddle coil with switchable sensitivity. The micro coil of the saddle-shaped mode does not have high sensitivity, but has a large sensitive area. On the other hand, since the shape of the micro coil of planar-shape mode is like a double-layered flat micro coil, it has a small but highly sensitive area.

Our concept of the micro saddle coil for local high-resolution MRI of luminal tissue is shown in Figure 2. The micro saddle coil consists of a flexible poly-imide substrate with coil patterns embedded in a polydimethylsiloxane (PDMS) tube. The micro coil was designed for detecting both small and large tumors in the luminal tissue such as the esophagus. The micro saddle coil has a flexible architecture and is deformable into a saddle-shaped mode and a planar-shaped mode.

The saddle-shaped mode has a large sensitive area, enabling large area MRI imaging of luminal tissue covering many tumors. The design of saddle-shaped mode is the commonly used saddle coil design of a length-to-diameter ratio of 2 and circular arcs of 120° [18]. Since the micro saddle coil has a thin hollow cylinder shape, the coil is easily deformed into the planar-shaped mode by pushing. By deforming into a flat planar shape, the sensitive area becomes concentrated at one side, and the sensitivity of the coil sufficiently increases to detect small tumors in luminal tissue. Thus, the micro saddle coil enabled large-area imaging and highly sensitive imaging by deforming its shape to switch the sensitive area and sensitivity of the coil.

### 2.2. Fabrication Process

**Figure 3:**
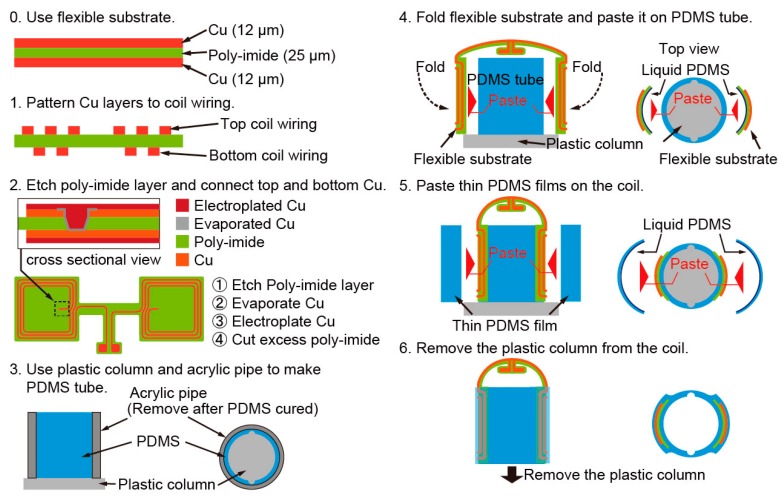
The fabrication process of the micro saddle coil.

Figure 3 shows the fabrication process of the micro saddle coil. We use the 25-μm-thick flexible poly-imide sheet with 12-μm-thick top and bottom Cu layers. First, the top Cu layer and the poly-imide layer were etched to make a through hole for connecting the top and bottom Cu layers. Then, the top and bottom Cu layers were etched to make the coil wiring. The top and bottom Cu layers were connected by evaporating the Cu and electroplating another Cu layer as shown in step 2 of Figure 3. The thickness of the Cu layers were 50 μm after electroplating. The excess poly-imide layer was cut away, and liquid PDMS was applied as a paste to the back of the flexible substrate. To make the PDMS tube, we pour liquid PDMS between the acrylic pipe and the plastic column with a bump. The flexible substrate was pasted onto the PDMS tube with the plastic column. A thin PDMS film was pasted onto the flexible substrate to prevent it from peeling off. Finally, the PDMS was full cured to fix the flexible substrate into the PDMS tube, and the plastic column was removed.

**Figure 4:**
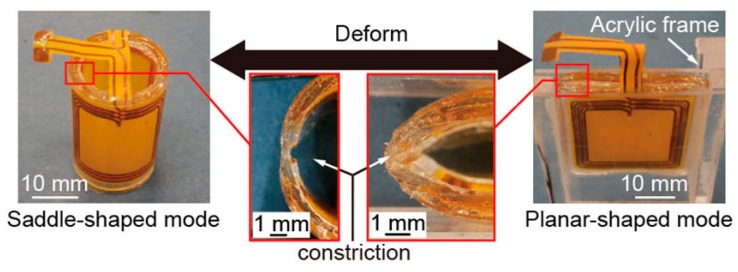
Photographs of the fabricated micro saddle coil. The coil deforms from saddle-shaped mode to planar-shaped mode. Since the polydimethylsiloxane (PDMS) tube has constricted parts, the coil deformations are reproducible.

Figure 4 shows the photograph of the fabricated micro saddle coil. The diameter and the length of the fabricated coil are 20 and 30 mm, respectively. The number of turns of the coil is 10, and the gap of the wiring is 600 μm. The width and thickness of the coil wiring are 500 and 50 μm, respectively. The coil of saddle-shaped mode deforms to planar-shaped mode by pushing. Since the PDMS tube has two constricted parts, the micro saddle coil easily deforms and the deformations are reproducible.

## 3. Experiment

### 3.1. Electrical Characteristics of the Coil

**Figure 5:**
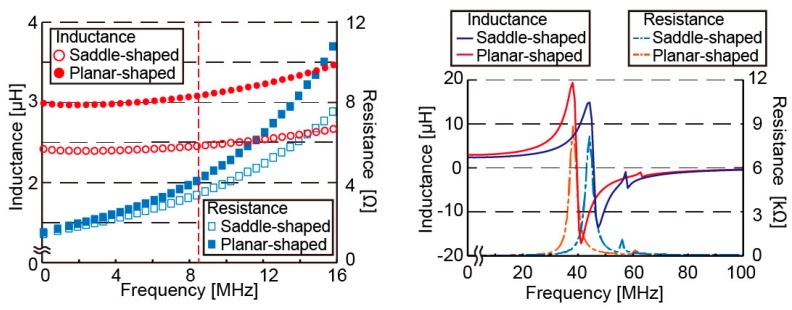
Characteristics of the micro saddle coil. The resistance and inductance of the coils is plotted from 1 to 16 MHz (**left**) and from 1 to 100 MHz (**right**).

We measured the electrical characteristics of the micro saddle coil in both saddle-shaped and planar-shaped modes. We use the open-type MRI that has the static magnetic field of 0.2 T and the MRI frequency of 8.5 MHz. The electrical characteristics of the micro saddle coil are presented in Figure 5 and Table 1. At 8.5 MHz, the inductance of the saddle-shaped and planar-shaped mode was 2.45 and 3.07 μH, respectively, and their respective resistance was 3.31 and 3.92 Ω. The Q-factor was 39.9 and 42.9, respectively. All three of the resistance, inductance, and Q-factor were increased. Since the Q-factor was increased, deformation into planar form increased the sensitivity of the micro saddle coil. The self-resonant frequency was higher in saddle-shaped mode than in the planar-shaped mode (44.8 MHz *vs.* 39.5 MHz). It can be concluded that coil deformation reduced the gaps between the coil wirings and increased the parasitic capacitance of the coil.

### 3.2. Experimental Setup for Taking MRI Images

**Figure 6:**
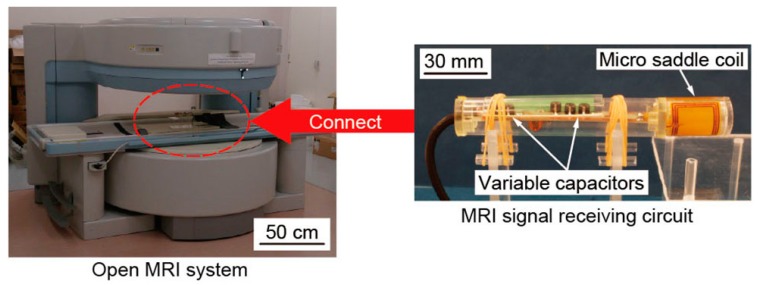
Experimental setup for measuring the MRI images. The micro saddle coil is attached to the receiving circuit, which is connected to the MRI system.

To evaluate the sensitivity and the sensitive area of fabricated micro saddle coil, we took MRI images by using the experimental setup shown in Figure 6. We used an open-type MRI system with 0.2-T static magnetic fields. Excitation coil is embedded in the MRI system together with the permanent magnets, and the fabricated coil received only the NMR signal. The coil was attached to an MRI signal receiving circuit comprising the micro saddle coil, the protection circuit, and two variable capacitors for tuning the frequency and matching the impedance of the circuit. The receiving circuit placed into the acrylic pipe was connected to an open-type medical MRI system. The receiving circuit and measurement object were placed at the center of the MRI system. As compared to the micro saddle coil, we have used a medical head coil, because the medical head coil is the smallest coil among the accompanying coils of the medical MRI system we used. The diameter and length of the medical head coil are about 300 and 350 mm. All MRI images were acquired under the following conditions: spin echo (SE) sequence with a repetition time (TR) and echo time (TE) of 250 and 22 ms, respectively, and a repeat count of 16 times. We took the MRI images of 0.5 × 0.5 × 1.0 mm^3^ and 2.0 × 2.0 × 2.0 mm^3^ spatial resolution. The field of view (FOV) and the acquisition matrix were 160 × 160 mm^2^ and 80 × 80 at the voxel size of 2.0 × 2.0 × 2.0 mm^3^. Those at the voxel size of 0.5 × 0.5 × 1.0 mm^3^ were 80 × 80 mm^2^ and 160 × 160.

### 3.3. SNR Measuring Experiment

**Figure 7:**
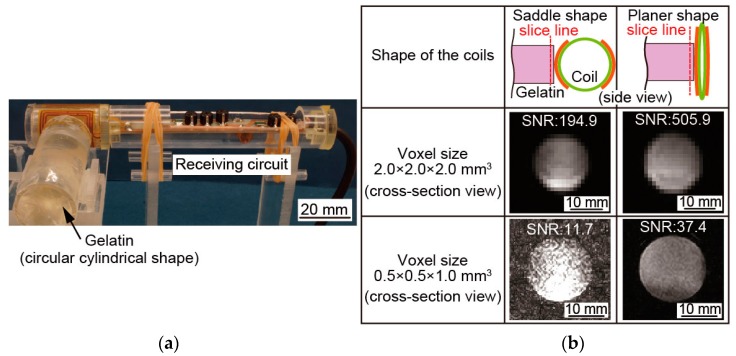
Experimental setup of signal-to-noise ratio (SNR) measurement and the taken MRI images of SNR measuring experiment. (**a**) Experimental setup; (**b**) MRI images of SNR measurement.

We measured the SNRs of the MRI images that were taken by saddle-shaped mode and planar-shaped mode. Figure 7a shows the experimental setup for measuring the SNRs of the micro saddle coil. To facilitate the measurement of the SNR in this experiment, we used a cylindrical gelatin as the reference measurement object. The MRI images of the gelatin were taken by the medical head coil and the micro saddle coil in saddle-shaped and planar-shaped modes. In the medical head coil, the gelatin was placed in the center. In saddle-shaped mode, the gelatin was placed at the side of the coil. In planar-shaped mode, the micro coil was pushed to the gelatin.

The MRI images taken by the SNR measurement experiment are shown in Figure 7b. We obtained a cylindrical cross section of the gelatin for the SNR measurement. Signal intensity of the signal area was a circle that best inscribed in each image. Signal intensity of the noise area was the average value of the region of the four corners in each image. Then, we calculated the SNR of the MRI image by dividing the signal intensity of the signal area by the signal intensity of the noise area.

Table 2 presents the SNRs of the medical and the micro saddle coil in two modes. The SNRs of the medical head coil were 41.7 and 3.5 at voxel sizes of 2.0 × 2.0 × 2.0 mm^3^ and 0.5 × 0.5 × 1.0 mm^3^, respectively. In contrast, the SNRs of the saddle-shaped and planar-shaped modes were 194.9 and 505.9 respectively at 2.0 × 2.0 × 2.0 mm^3^, and 11.7 and 37.4 respectively at 0.5 × 0.5 × 1.0 mm^3^. The SNRs of the saddle-shaped modes were improved by 259% at a voxel size of 2.0 × 2.0 × 2.0 mm^3^, and by 319% at 0.5 × 0.5 × 1.0 mm^3^ as compared to the SNRs of the planar-shaped mode.

### 3.4. Visible Area Measuring Experiment

**Figure 8:**
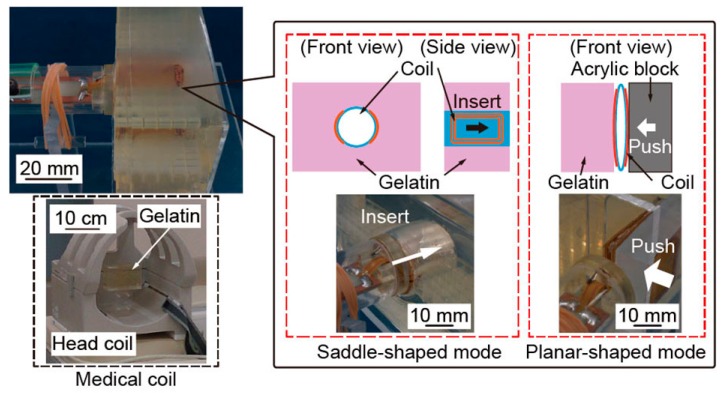
Schematic of the gelatin placement. The micro saddle coil was inserted into the gelatin in saddle-shaped mode, and pushed against the gelatin in planar-shaped mode. The medical head coil is evaluated for comparison.

**Figure 9:**
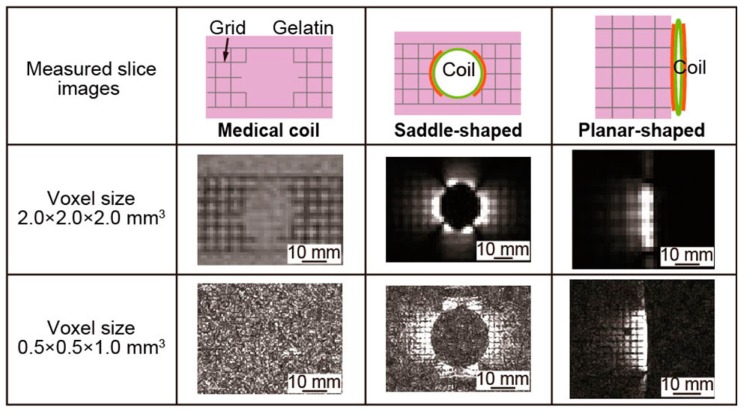
The MRI images taken by medical head coil, saddle-shaped, and planar-shaped mode coil with the voxel size of 0.5 × 0.5 × 1.0 mm^3^ and 2.0 × 2.0 × 2.0 mm^3^.

To evaluate the size of the sensitive area, we took the MRI images of gelatin containing an inserted grid. Figure 8 shows the schematics of the experimental setup of visible area measurement. In the saddle-shaped mode, the micro saddle coil was inserted into the gelatin. In planar-shaped mode, the micro saddle coil was pushed against the gelatin. We also measured the gelatin by the medical head coil for comparison. The measured MRI images are shown in Figure 9.

As shown in Figure 9, the sensitive area of the medical head coil was large, but the MRI image was excessively noisy at the small voxel size of 0.5 × 0.5 × 1.0 mm^3^. The saddle-shaped mode admits a large sensitive area around the coil. On the other hand, the sensitive area of the planar-shaped mode was small, but clear MRI images were taken.

**Figure 10:**
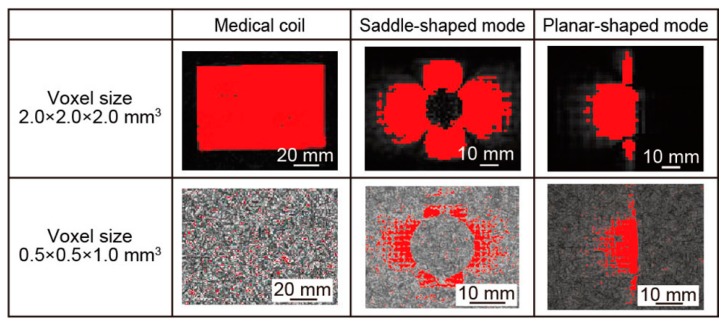
The visible area of the MRI images of medical head coil, saddle-shaped coil, and planar-shaped mode coils with the voxel size of 0.5 × 0.5 × 1.0 mm^3^ and 2.0 × 2.0 × 2.0 mm^3^. Red pixels show the visible area of the coil.

To evaluate the MRI images quantitatively, we used the image analysis software to calculate the visible area. The measured MRI images were converted to the images with 256 stage of brightness. We used the four corners of the converted image as a noise area. The visible area at voxel size of 2.0 × 2.0 × 2.0 mm^3^ was defined as the area with more than 8 times the brightness of the noise area. The visible area at voxel size of 0.5 × 0.5 × 1.0 mm^3^ was defined as the area with more than 3 times the brightness of the noise area. As shown in Figure 10, the red area is the visible area of the MRI image. At this time, the sizes of 1 pixel are 2.0 × 2.0 mm^2^ and 0.5 × 0.5 mm^2^ at the voxel size of 2.0 × 2.0 × 2.0 mm^3^ and 0.5 × 0.5 × 1.0 mm^3^, respectively. We counted the red pixels of each image and calculated the visible area of the MRI images. Table 3 shows the size of the visible area. In the voxel size of 2.0 × 2.0 × 2.0 mm^3^, the visible area of the medical head coil, the saddle-shaped mode, and the planar-shaped mode were 37,268, 5188, and 1196 mm^2^, respectively. In the voxel size of 0.5 × 0.5 × 1.0 mm^3^, the visible area of the medical head coil, the saddle-shaped mode, and the planar-shaped mode were 41.5, 987.5, and 320.3 mm^2^, respectively. We can indicate the visible area in each image quantitatively.

## 4. Discussion

### 4.1. SNR Improvement of the MRI Images Taken by Micro Saddle Coil

As shown in Figure 7 and Table 2, the SNRs of the planar-shaped mode were about 10 times larger than the SNRs of medical head coil. The SNRs of the planar-shaped mode were about 3 times larger than those of the saddle-shaped mode. This nearly threefold improvement in the SNR at both voxel sizes confirms the high sensitivity of our coil, achieved by deforming and concentrating the sensitive area. This improvement of the sensitivity is considered because the increase in number of turns per unit length and the overlap of the sensitive area.

The saddle-shaped mode has a sensitive area inside of the coil as well. Since this sensitive area overlaps due to the deformation, the sensitivity is considered to be nearly doubly improved. However, the actual SNRs are improved by nearly three times. This improvement of SNRs can be explained by the increase in the number of turns per unit length.

From the above, we can improve the sensitivity of the coil and take high sensitive MRI images by deforming the shape of the micro saddle coil. Moreover, the micro saddle coil is able to take MRI images in a resolution that is difficult to achieve with medical MRI.

On the other hand, since the inductance of the micro coil was changed with the deformation, the impedance of the receiving circuit could match to MRI in only one mode. Therefore, the deformable coil with the mechanism to avoid the inductance change was studied [19]. By adding an automatically tuning mechanism, it is expected that the impedance of the receiving circuit would match in both modes.

### 4.2. Comparison of Visible Area of MRI Images

As shown in Figure 10, the visible area of the medical head coil at the voxel size of 2.0 × 2.0 × 2.0 mm^3^ is 37,268 mm^2^. Although the obtained MRI image is slightly blurred, most of gelatin area can be visualized by the medical head coil. At the voxel size of 0.5 × 0.5 × 1.0 mm^3^, we cannot observe the gelatin and grid from the MRI image taken with the medical head coil. On the other hand, we can observe the gelatin and grid from the MRI image of the micro saddle coil in two modes not only at the voxel size of 2.0 × 2.0 × 2.0 mm^3^ but also at the voxel size of 0.5 × 0.5 × 1.0 mm^3^. The visible area of the saddle-shaped and planar-shaped modes are 987.5 mm^2^ and 320.3 mm^2^ at the voxel size of 0.5 × 0.5 × 1.0 mm^3^. Therefore, we confirm that the micro saddle coil is capable for measuring the MRI images at the voxel size that has been difficult for the medical head coil.

Comparing the saddle-shaped mode with the planar-shaped mode, the saddle-shaped mode can take MRI images of a large area around the cylinder. The planar-shaped mode can take MRI images of a small semicircular area adjacent to the coil. The size of the visible area of the saddle-shaped mode was about 4.3 times larger than that of the planar-shaped mode at a voxel size of 2.0 × 2.0 × 2.0 mm^3^, and about 3.0 times larger at a voxel size of 0.5 × 0.5 × 1.0 mm^3^. Since the saddle-shaped mode has a visible area not only on the right and left sides of the coil but also the top and bottom sides of the coil, the MRI images of the saddle-shape mode is considerably larger.

**Figure 11:**
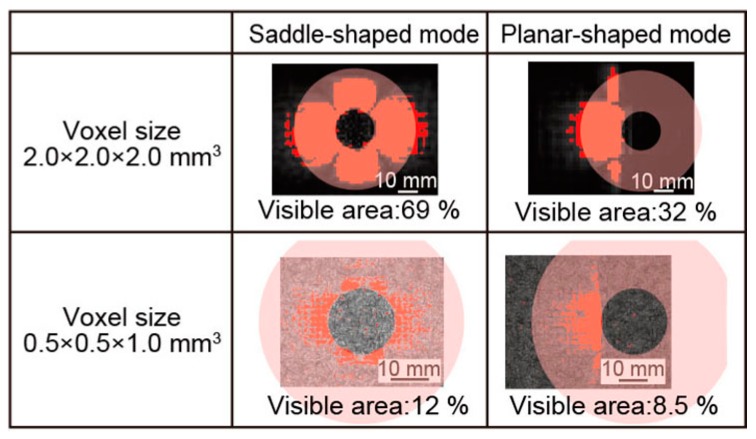
The image area in the case of repeated lumen tissue.

Finally, we evaluated the micro saddle coil using the ratio of the visible area to the cross-sectional area of the esophagus. We defined the hollow cylinder with the diameter of 60 mm as the cross-sectional area of the esophagus. Figure 11 shows the comparison results of the visible area to the cross-sectional area of esophagus. The ratio of the visible area of the saddle-shaped and the planar-shaped modes were 68% and 32% at the voxel size of 2.0 × 2.0 × 2.0 mm^3^, respectively. The ratio of the visible area of the saddle-shaped and the planar-shaped modes were 12% and 8.5% at the voxel size of 0.5 × 0.5 × 1.0 mm^3^.

Since the saddle-shaped mode could visualize approximately 70% area of esophagus at the voxel size of 2.0 × 2.0 × 2.0 mm^3^, it was expected that a small tumor in deep luminal tissue would be detected by single imaging of the saddle-shaped mode. On the other hand, the planar-shaped mode had only 8.5% area of esophagus at the voxel size of 0.5 × 0.5 × 1.0 mm^3^. Although the visible area was very small, the SNRs of MRI images were very high, and the clear MRI images of high resolution could be acquired by the planar-shaped mode. Therefore, it was expected that a very small tumor would be detected by changing the shape of the micro saddle coil to the planar-shaped mode.

## 5. Conclusions

In conclusion, we fabricated a micro saddle coil with switchable sensitivity for MRI. The diameter and length of the coil are 20 and 30 mm, respectively, and 10 turns of wiring are used. Since the coil is embedded in the PDMS tube, the micro saddle coil can deform from saddle-shaped mode to planar-shaped mode via pushing.

As a result of measuring the electrical characteristics of the micro saddle coil at 8.5 MHz, the inductance, the resistance, and the Q-factor of the saddle-shaped mode were 2.45 μH, 3.31 Ω, and 39.9, respectively. Those of the planar-shaped mode were 3.07 μH, 3.92 Ω, and 42.9, respectively. The self-resonant frequencies of the saddle-shaped and planar-shaped mode were 44.8 MHz and 39.5 MHz. Therefore, the electrical characteristics of the micro saddle coil in two modes were high enough for using the coil as a MRI signal receiver.

We attached the fabricated micro saddle coil to a MRI signal receiving circuit, and connected to an open-type medical MRI system. We took MRI images for evaluating the sensitivity and the range of the sensitive area. The saddle-shaped mode had the SNR of 194.9 and sensitive area of 5188 mm^2^ at the voxel size of 2.0 × 2.0 × 2.0 mm^3^. The saddle-shaped mode had the large visible area around the coil and could be observed almost 70% area of the cross-sectional area of esophagus. At the voxel size of 0.5 × 0.5 × 1.0 mm^3^, the SNR and sensitive area were 11.7 and 987.5 mm^2^. We were barely able to observe the MRI image around the coil. On the other hand, the planar-shaped mode had the SNR of 505.9 and sensitive area of 1196 mm^2^ at the voxel size of 2.0 × 2.0 × 2.0 mm^3^. At the voxel size of 0.5 × 0.5 × 1.0 mm^3^, the SNR and sensitive area were 37.4 and 320.3 mm^2^. The sensitive area was much reduced in planar-shaped mode, but this mode had very high SNRs and ensured clear MRI images.

Since our coil concentrates the sensitive area to achieve high sensitivity, the sensitivity of the saddle-shaped and the planar-shaped modes were about 3 times and 10 times higher than those of the medical head coil at both voxel sizes. Thus, by switching the coil sensitivity, we acquired MRI images over a large area and over a small area with greater sensitivity.

## Figures and Tables

**Table 1:** Electrical characteristics of the coil at 8.5 MHz.

Coil Shape	Saddle-Shaped Mode	Planar-Shaped Mode
Inductance (μH)	2.45	3.07
Resistance (Ω)	3.31	3.92
Q-factor (–)	39.9	42.9
Self-resonant frequency (MHz)	44.8	39.5

**Table 2:** The SNR of the medical head coil and micro saddle coil.

Voxel Size	SNRs of MRI Images			Increase Rate from Saddle-Shape to Planar-Shape
	Medical Head Coil	Saddle-Shaped Mode	Planar-Shaped Mode	
2.0 × 2.0 × 2.0 (mm^3^)	41.7	194.9	505.9	259%
0.5 × 0.5 × 1.0 (mm^3^)	3.5	11.7	37.4	319%

**Table 3:** The size of the visible area of the medical and micro saddle coil.

Voxel Size	Medical Head Coil	Saddle-Shaped Mode	Planar-Shaped Mode
2.0 × 2.0 × 2.0 (mm^3^)	37,268 mm^2^	5188 mm^2^	1196 mm^2^
0.5 × 0.5 × 1.0 (mm^3^)	41.5 mm^2^	987.5 mm^2^	320.3 mm^2^
